# 

**Published:** 1800-02

**Authors:** 


NEW MEDICAL PUBLICATIONS IN JANUARY.

An Inquiry into the Symptoms and Caufes of Syncope Anginofa, commonly called Angina Pectoris; illuftrated by Diffections. By CALEB HILIER PARRY, M. D. &c. 4s. Cadell and Davies.

The Anatomy of the Gravid Uterus, with practical inferrences relative to Pregnancy and Labour. By JOHN BURNS, Surgeon in Glafgow, 8vo. pp. 248. 5s. Longman and Rees.

Obfervations on the Difeafes of Seamen. By GILBERT BLANE, M. D. &c. &c. The third edition, with corrections and additions, 8vo. p. 626. 9s. Murray and Highley.

A Treatife on Sugar, with mifcellaneous Medical Obfervations. By BENJAMIN MOSELY, M. D. &c. 8vo. 6s. 6d. Robinfons.

Some few Cafes and Obfervations on the Treatment of Fiftula in Ano, Hæmorrhage, Mortification, the Venereal Difeafe, and Strictures of the Urethra. By JOHN ANDREE, M. D. &c. 1s. 6d. G. and W. Nicol.

The Anatomifts Vade Mecum, containing the Anatomy and Phyfiology of the Human Body. By ROBERT HOOPER, M. D. F. L. S. &c. fecond edition, revifed and enlarged, 3s. 6d. Murray and Highley.

A Letter to Mr. Ogden, on the Cæfarean Operation. By G. TOMLINSON, 8vo. pp. 20. 1s. *Manchefter*, I. and W. Clarke.

NEW MEDICAL PUBLICATIONS IN GERMANY.

[ Imported by C. Geifweiler, No. 42, Parliament Street. ]

De Penitiori Offium Structura Commentarius, Auctore ANTONIO SCARPA, in ticinenfi gymnafio anatomes et chirurgiae clinices profeffore, &c. &c. gr. 4to. vellum paper, hot preffed, cuts, 14s. 1799. *Leipzig.*

De Morbis Ligamentorum ex materiei animalis mixtura, et Structura mutata cognofcendis, Auctor GODOFREDUS GOETZ, Gedanenfis, cum obfervatione Mecrelii, 4to. 5s. cuts, fewed, 1799. *Berlin.*

MARTIN CHRISTIAN GOTTLIEB LEHMANN, Holfati, Seminari Regii Philologici Sodalis; de Senfibus externis Animalium exfanguium Infectorum fcilicet ac Vermium Comentatio, in certamine literario civium Academiæ Georgiæ Auguftæ, præmio a rege M. Britaniæ aug. conftituto, ab illuftri Medicorum ordine ornata, 4to. 2s. 6d. fewed, 1799. Göttingen.

[ *The lift of French publications in our next.* ]

### TO CORRESPONDENTS.

The Extracts mentioned by Dr. Harrington have not been received.

Mr. Martineau's Communication has been mislaid, and we fhall be obliged to him for a Copy of it.

The numerous Applications refpecting the moft approved Medical Thermometers will be anfwered in our next.

Mr. Watt's Improvement of Frere Coom's Inftrument is received.

